# Rubeola

**Published:** 1873-02-15

**Authors:** N. S. Davis

**Affiliations:** Professor of Clinical Medicine, Etc.


### Clinical Lectures.

### RUBEOLA.

LECTURE DELIVERED IN MEDICAL WARDS OF MERCY HOSPITAL. BY N. S. DAVIS, M.D., PROFESSOR OF CLINICAL MEDICINE, ETC.

Gentlemen:—The girl lying in this bed was taken sick some five or six days ago, and was brought to the Hospital on yesterday.

On examining her, you will see that she presents the characteristic eruption of measles; easily distinguished from that occurring in scarlatina by the points of eruption being aggregated in clusters, with natural skin between; and from that of small-pox, in which the eruption comes in pointed, elevated, hard nubs, not so red as in this case, presenting only a slight flush, and without the catarrhal premonitory symptoms, or weeping of the eyes. The patient is now at the stage when she is suffering oppression and tightness of the chest, with a harsh cough, which produces intense pain through the temples with each paroxysm.

In this disease, if uncomplicated, the indications for treatment are very plain and simple. The disease has a natural course to run; cannot be broken up; is self-limited; and the physician is not expected to interfere with active means, but simply to modify its severity as much as possible, so as to leave the system in the best possible condition.

Give enough of some anodyne expectorant to lessen the severity of the bronchial irritation and cough, and mitigate the pain in the head. One of the best preparations, perhaps, for cases as severe as this, consists in the following: ℞.—Syrup scillæ comp., ℥ iss.; vinum antimonii, ℥ ss.; tinct. opii camp., ℥ ij.; tinct. verat. viride, ʒ i.—M. Dose—One teaspoonful every three hours, in a tablespoonful of water.

This will, usually, in the course of twenty-four hours, lessen the fever and modify the cough, while the pain in the head will at the same time be greatly relieved.

As the period is reached when the fever begins to decline, the veratrum may be omitted from the mixture, which may then be continued, given every three, four, or five hours, till the cough has entirely disappeared. The anodyne, with the expectorant, produces a very pleasant effect, and does not tend to interfere with the progress of the eruption; but you will usually find, while using this, that the bowels will become constipated, tongue coated, urine scanty, etc., which condition, if neglected, leads to a bad state of digestion, disorder of the bowels, etc.

I am not in the habit of giving physic until the eruption is fairly out; when, if the bowels have not moved for a couple of days, I direct a mild laxative, as, for instance, a combination like the following: ℞.—Hydrarg. chl. m., grs. v.; leptandrin, grs. ij.; soda bicarb., grs. v.—M. This will produce a moderately fair operation of the bowels, which may subsequently be kept in a regular condition by any simple laxative that will be most readily taken: Rochelle salts, effervescing solution, Tarrant's aperient, pil rhei comp., etc.

You will occasionally meet with cases where the patient suffers so much at night that it is best to give a tolerably full dose of Dover's powder at bed-time. A good combination for this purpose is, pulv. Doveri, vii. grs., with hydrarg. chl. mit., i gr., followed, if necessary, by a laxative in the morning. Or, you may use instead, fifteen or twenty grains of the bromide of potassium at night. This sometimes acts very favorably, but is not reliable in cases of eruptive fever.

One important thing to guard against is extension of the irritation from the bronchial tubes to the lobules of the lungs, making it complicated with lobular pneumonia.

In children under two years of age this tendency is very strong.

It is usually about the second day of the eruption that you will first be able to detect this, if it is going to occur; it may happen later; seldom, however, before the second or third day.

You will observe, first, that they do not breathe naturally, nor as from simple tightness of the bronchial tubes ; but every inspiration brings out a forcible expansion of the nostrils, and at the beginning of the expiratory act there is sudden falling in of the walls of the abdomen, produced by contraction of the abdominal muscles.

These, if the case be noticed carefully, will be among the earliest symptoms to warn you of trouble with the lungs. On applying the ear to the chest, you will find a sharp, well-defined subcrepitant rhoncus.

If allowed to run along till the time for the fever to subside, you will have the little lobules of the lungs in a hepatized condition; the patient drowsy and dull; breathing short; lips blue; pulse sharp, short, and quick in stroke, and easily compressed; lips and tongue dry; patient disposed to lay with the head thrown back, eyes half open; and in a few days death will supervene.

Pneumonia is the chief source of fatality from measles.

When symptoms of pneumonia occur, in connection with measles, the best remedy in children is a combination like the following: ℞.—Liq. ammon. acetas, ℥ iss.; syrup ipecac, ℥ ss.; tinct. opii et camph., ℥ i.; tinct. verat. viride, ʒ i. The dose proportioned to the age of the child. For a child two years old, about twenty drops are usually necessary; though it is best to begin with ten drops. For an adult, one teaspoonful. It should be given every two, three, or four hours, till the fever is controlled. In the active stage of the disease, while using this mixture, cover the chest externally with fomentations.

I have considerable faith in the popular notion about onions. They certainly afford more relief to the breathing than any other thing we can use. I attribute it to the impregnation of the air which is inhaled with the volatile oil, more than to any absorption from the surface of the chest, and think this application preferable to blistering.

It will be advisable, in these cases, to give a powder, containing from half a grain to a grain of calomel, with Dover's powder, according to the age and restlessness of the patient, about three times a day. The liquid mixture may be continued, at longer intervals, until the symptoms of pneumonia have entirely disappeared.

